# Biodiversity inventories and conservation of the marine fishes of Bootless Bay, Papua New Guinea

**DOI:** 10.1186/1472-6785-12-15

**Published:** 2012-08-01

**Authors:** Joshua A Drew, Charlene L Buxman, Darcae D Holmes, Joanna L Mandecki, Augustine J Mungkaje, Amber C Richardson, Mark W Westneat

**Affiliations:** 1 Department of Zoology and Biodiversity Synthesis Center, Field Museum of Natural History, 1400 South Lake Shore Drive, Chicago, IL 60605, USA; 2 Department of Organismal Biology and Anatomy, The University of Chicago, 1027 E 57th St, Chicago, IL, 60637, USA; 3 VOISE Academy High School, Chicago Public Schools, 231 North Pine Avenue, Chicago, IL, 60644, USA; 4 Motupore Island Research Center, University of Papua New Guinea, P.O. Box 320 University Post Office, Port Moresby, National Capital District, Papua New Guinea; 5 Current Address: Department of Ecology, Evolution and Environmental Biology, Columbia University, 1200 Amsterdam Ave, New York, NY 10027, USA

**Keywords:** Collections-based research, Shifting baselines, Biodiversity inventories, Coral reef ecosystems, Biodiversity informatics

## Abstract

**Background:**

The effective management and conservation of biodiversity is predicated on clearly defined conservation targets. Species number is frequently used as a metric for conservation prioritization and monitoring changes in ecosystem health. We conducted a series of synoptic surveys focusing on the fishes of the Bootless Bay region of Papua New Guinea to generate a checklist of fishes of the region. Bootless Bay lies directly south of Port Moresby, the capital of Papua New Guinea, and experiences the highest human population density of any marine area in the country. Our checklist will set a baseline against which future environmental changes can be tracked.

**Results:**

We generated a checklist of 488 fish species in 72 families found in Bootless Bay during a two-week sampling effort. Using incident-based methods of species estimation, we extrapolate there to be approximately 940 fish species in Bootless Bay, one of the lowest reported numbers in Papua New Guinea.

**Conclusions:**

Our data suggest that the Bootless Bay ecosystem of Papua New Guinea, while diverse in absolute terms, has lower fish biodiversity compared to other shallow marine areas within the country. These differences in faunal diversity are most likely a combination of unequal sampling effort as well as biophysical factors within Bootless Bay compounded by historical and/or contemporary anthropogenic disturbances.

## Background

Understanding the magnitude and direction of ecosystem change requires careful documentation of thespecies present within that ecosystem. Without quantitative data, large-scale changes in one generation can be overlooked, resulting in a gradual shift towards increasingly degraded natural states being accepted as the baseline for future comparisons [1]. The reefs of Papua New Guinea are some of the most diverse in the world and are part of a region dubbed the “Coral Triangle,” an area bounded by the Philippines, Papua New Guinea and Indonesia. The Coral Triangle is the epicenter of marine biodiversity [2,3] for numerous taxonomic groups including fishes [4], snails [5] and lobsters [3]. Papua New Guinea’s approximately 14,535 km^2^ of reefs represent 6% of the world’s reefs. Over 50% of Papua New Guinea’s reefs are currently threatened, a number that may rise to an estimated 78% when models of increasing thermal stress are incorporated [6]. Only 4% of Papua New Guinea’s reefs are within officially designated marine protected areas [6], and while alternative conservation measures (such as traditional closures [7,8]) do exist, there is a real potential for the faunal and structural composition of Papua New Guinea to be substantially altered in the timescale of a single human generation.

Bootless Bay is a semi-enclosed bay on the southwest coast of Papua New Guinea. The bay is approximately 9.5 km along its longest axis (northwest-southeast) and 2 km wide. The bay is shallow with a maximum depth of approximately 30 m. The main interface with the Pacific Ocean is on the southwest side. There are no major rivers emptying into the bay but several small creeks do provide a constant freshwater influx. The surrounding vegetation is largely savannah with a *Themeda triandra* understory beneath *Eucalyptus spp.* canopy [9]. There is also a small mangrove restoration project in the northeast portion of the Bay.

**Figure 1:**
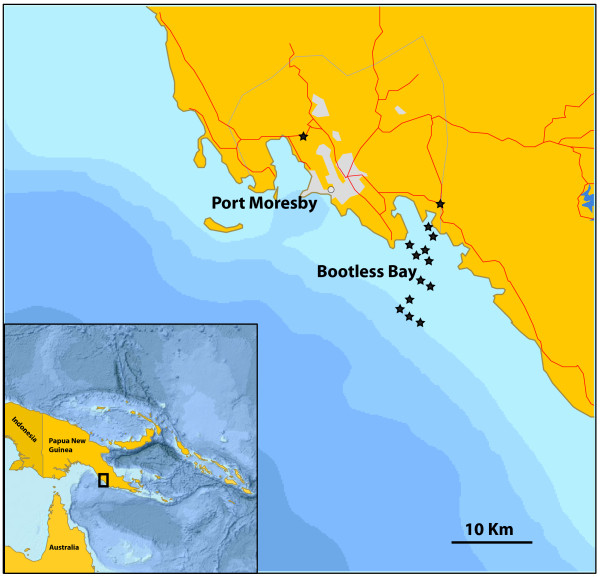
**Map of study area.** Approximate sampling locations indicated by a star. Actual latitude and longitude for sampling locations are given in Table 1.

Port Moresby is the capital and major population center of Papua New Guinea and is located approximately 10 km northwest of Bootless Bay (Figure 1). Population pressure is one of the major threats facing the reefs in Papua New Guinea. In part due to their proximity to the markets in Port Moresby [8], we would expect Bootless Bay reefs to experience greater levels of degradation than more remote reefs [6,8]. Additional threats may include unsustainable fishing for foreign markets, habitat degradation and sedimentation through upstream land practices [10]. Before quantifying the degree of environmental change potentially caused by anthropogenic or other stressors, we need to establish a baseline of fish biodiversity to which future sampling can later be compared.

In this paper we use a combination of empirical field sampling, literature review and collections-based research to compile a checklist of the fishes found in Bootless Bay. This paper, in conjunction with the collections in natural history museums, will serve as a baseline of fish diversity found in Bootless Bay in the early 21^st^ century.

## Results

### Species number and accumulation

**Figure 2:**
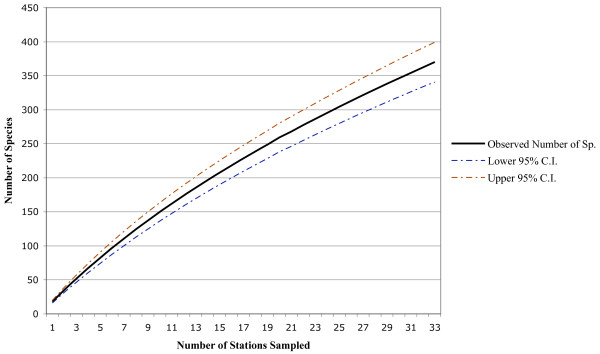
**Species accumulation curve based on sampling effort outlined in Table**1.

**Figure 3:**
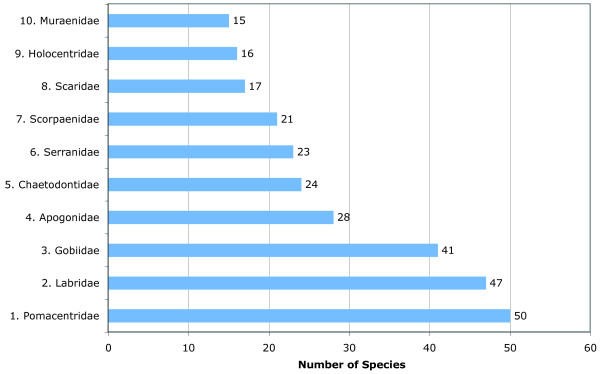
**Ten most species-rich families based on Table**2.

Sampling 33 stations over a two-week period yielded 384 fish species with a mean of 23.32 individuals representing 19.36 species collected per site (Table 1). The species accumulation curve (Figure 2) showed a continued increase in species per station indicating that additional sampling in the area is likely to provide new records. The confidence intervals for our species accumulation curve suggest a species richness between 340 and 399 species. The inclusion of other records from published literature and museum collections added another 101 species, bringing the total in this preliminary checklist to 485 species representing 72 families (Table 2). The ten most species-rich families accounted for 57.5% of the total number of identified species (Figure 3).

**Table 1:** List of location, collecting methodology, habitat type and numbers of individuals and species recorded for all collecting stations.

**Station**	**Latitude**	**Longitude**	**Collecting methodology**	**Habitat**	**Number of individuals**	**Number of species**
PNG 11-01	09° 28.674’ S	147° 11.968’ E	Fish Market - hook and line, spear	City market	9	12
PNG 11-02	09° 31.322’ S	147° 17.076’ E	Spear (Hawaiian sling), snorkeling	Seagrass bed, rocky intertidal	1	1
PNG 11-03	09° 32.085’ S	147° 16.619’ E	SCUBA, Rotenone (1kg powder), spear, hook and line	Coral Reef	56	45
PNG 11-04	09° 52.434’ S	147° 16.583’ E	SCUBA, spear, hook and lane	Coral Reef	26	25
PNG 11-05	09° 31.322’ S	147° 17.076’ E	Snorkel, spear (small Filipino gun and Hawaiian sling)	Seagrass, patch reef, rocks	22	17
PNG 11-06	09° 31.322’ S	147° 17.076’ E	Dip net, dive light, walking around reef flat	Sandbar	3	3
PNG 11-07	09° 28.674’ S	147° 11.968’ E	Hook and line, spear	City Market	11	11
PNG 11-08	09° 30.447’ S	147° 17.209’ E	Gill net	Fishers at Dock	1	1
PNG 11-09	09° 35.625’ S	147° 17.021’ E	SCUBA, Rotenone (1kg powder), spear, hook and line	Coral Reef	35	32
PNG 11-10	09° 35.625’ S	147° 17.021’ E	Spear (small Filipino gun and Hawaiian sling)	Coral Reef	50	43
PNG 11-11	09° 34.233’ S	147° 17.286’ E	SCUBA, Rotenone (1kg powder), spear, hook and line	Coral Reef	74	53
PNG 11-12	09° 34.233’ S	147° 17.286’ E	Spear (small Filipino gun and Hawaiian sling)	Coral Reef	50	31
PNG 11-13	09° 31.322’ S	147° 17.076’ E	Hand net	Beach- sand and coral rock	1	1
PNG 11-14	09° 32.673’ S	147° 16.494’ E	SCUBA, Rotenone (1kg powder), spear, hook and line	Coral Reef	45	40
PNG 11-15	09° 32.343’ S	147° 16.087’ E	Spear (small Filipino gun and Hawaiian sling), small mesh gill net	Coral Reef	44	33
PNG 11-16	09° 28.674’ S	147° 11.968’ E	Hook and line, spear	Citymarket	19	17
PNG 11-17	09° 31.322’ S	147° 17.076’ E	Spear (small Filipino gun and Hawaiian sling)	Reef, seagrass	5	5
PNG 11-18	09° 56.219’ S	147° 17.803’ E	SCUBA, Rotenone (1kg powder), spear, hook and line	Coral Reef	37	35
PNG 11-19	9° 36.319’ S	147° 17.803’ E	Spear (small Filipino gun and Hawaiian sling)	Coral Reef	31	28
PNG 11-20	9° 31.322’ S	147° 17.076’ E	Hook and line	Reef, seagrass	6	3
PNG 11-21	9° 30.003’ S	147° 17.542’ E	Rotenone (0.5 kg) and dipnets	Mangrove, river	18	18
PNG 11-22	9° 31.495’ S	147° 17.044’ E	Hand caught	Rocky shore, Mangrove	1	1
PNG 11-23	9° 32.214’ S	147° 16.469’ E	Spear (Hawaiian slings)	Coralreef	23	17
PNG 11-24	9° 30.983’ S	147° 16.918’ E	Spear (Hawaiian slings)	Coral reef	21	17
PNG 11-25	09° 31.322’ S	147° 17.076’ E	Handline with small hooks, baited with bread	Beach, rocky shore	3	1
PNG 11-26	09° 32.35’ S	147° 15.759’ E	SCUBA, rotenone, spear	Coral reef	69	65
PNG 11-27	09° 31.495’ S	147° 17.044’ E	Hand caught	Rocky shore, Mangrove	1	1
PNG 11-28	09° 31.322’ S	147° 17.076’ E	Hook and line	Beach, rocky shore	6	6
FNG 11-29	09° 31.495’ S	147° 17.044’ E	Rotenone (1 kg powder) and hand nets	Rocky shore, seagrass bed	51	34
PNG 11-30	09° 34.206’ S	147° 17.190’ E	Spear (small Filipino gun and Hawaiian sling)	Coral Reef	23	18
PNG 11-31	09° 34.206’ S	147° 17.190’ E	Spear (small Filipino gun and Hawaiian sling)	Coral Reef	26	20
PNG 11-32	09° 35.973’ S	147° 17.330’ E	Spear (small Filipino gun and Hawaiian sling)	Coral reef	2	2
PNG 11-33	09° 31.322’ S	147° 17.076’ E	Dip net, dive light, walking around reef flat	Sandbar	3	3
				Average	23.42	19.36
				Standard Deviation	21.46	17.29

**Table 2:** A list of marine fish species identified as occurring in Bootless Bay, Papua New Guinea

**Class**	**Order**	**Family**	**Genus + Species**
Chondrichthyes			
	Orectolobiformes		
		Stegostomatidae	
			***Stegostoma fasciatum *(Hermann 1783)**
		Hemiscyllidae	
			*Hemiscyllium hallstromi* Whitley, 1967
		Orectolobidae	
			***Eucrossorhinus dasypogon *(Bleeker 1867)**
	Carcharhiniformes		
		Carcharhinidae	
			*Carcharhinus melanopterus* (Quoy and Baimard, 1824)
			***Triaenodon obesus *(Rüppell 1837)**
	Rajiformes		
		Dasyatidae	
			*Dasyatis kuhlii* (Muller and Henle, 1841)
			*Taeniura lymma* (Bennett, 1830)
	Myliobatidae		
			*Aetobatis narinari* (Euphrasen 1790)
		Mobulidae	
			***Manta birostris *(Walbaum, 1792)**
Osteichthyes			
	Elopiformes		
		Megalopidae	
	Anguilliformes		*Megalops cyprinoides* (Broussonet, 1782)
		Congridae	
			*Heteroconger hassi* (Klausewitz & Eibl-Eibesfeldt, 1959)
			Congridae sp.
		Ophichthidae	
			***Callechelys marmorata *(Bleeker, 1853)**
			Kaupichthys sp.
			***Ophichthus bonaparti *(Kaup, 1856)**
		Anguilladae	
			*Anguilla obscura* Günther, 1872
		Muraenidae	
			*Echidna nebulosa* (Ahl, 1789)
			***Gymnothorax cf. chilospilus *Bleeker, 1864**
			*Gymnothorax elegans* Bliss, 1883
			***Gymnothorax favagineus *Bloch & Schneider, 1801**
			*Gymnothorax fimbriatus* (Bennett, 1832)
			***Gymnothorax flavimarginatus *(Rüppell, 1830)**
			*Gymnothorax herrei* Beebe & Tee-Van, 1933
			***Gymnothorax javanicus *(Bleeker, 1859)**
			*Gymnothorax richardsoni* (Bleeker, 1852)
			***Gymnothorax thyrsoidea *(Richardson, 1845)**
			*Gymnothorax undulatus* (Lacépède, 1803)
			Gymnothorax zonipectis Seale, 1906
			*Moringua* sp.
			*Pseudoechidna brummeri* (Bleeker, 1859)
			***Rhinomuraena quaesita *Garman, 1888**
	Clupeiformes		
		Clupeidae	
			Clupeidae sp.
	Siluriformes		
		Plotosidae	
			***Plotosus lineatus *(Thunberg, 1787)**
	Aulopiformes		
		Synodontidae	
			***Saurida gracilis *(Quoy & Gaimard 1824)**
			***Synodus dermatogenys *Fowler, 1912**
			***Synodus rubromarmoratus *Russell & Cressey 1979**
			*Synodus variegatus* (Lacépède 1803)
	Lophiiformes		
		Antennariidae	
			***Antennarius pictus *(Shaw, 1794)**
			***Histrio histrio *(Linnaeus, 1758)**
	Mugiliformes		
		Mugilidae	
			*Moolgarda seheli* (Forsskål, 1775)
	Beloniformes		
		Belonidae	
			*Tylosurus crocodilus* (Péron & Lesueur, 1821)
			***Zenarchopterus gilli *Smith 1945**
		Hemiramphidae	
			*Hemiramphus archipelagicus* Collette & Parin 1978
			*Hemiramphus far* (Forsskål, 1775)
			***Hyporhamphus quoyi *(Valenciennes, 1847)**
	Beryciformes		
		Holocentridae	
			*Myripristis berndti* Jordan and Evermann, 1903
			*Myripristis kuntee* Valenciennes, 1831
			*Myripristis murdjan* (Forsskål, 1775)
			***Myripristis violacea *Bleeker, 1851**
			***Myripristis vittata *Valenciennes, 1831**
			*Neoniphon argenteus* (Valenciennes, 1831)
			*Neoniphon sammara* (Forsskål, 1775)
			*Plectrypops lima* (Valenciennes, 1831)
			***Sargocentron caudimaculatum *(Rüppell 1838)**
			*Sargocentron cf. iota* Randall 1998
			*Sargocentron cornutum* (Bleeker 1853)
			***Sargocentron ensifer *(Jordan & Evermann 1903)**
			*Sargocentron rubrum* (Forsskål, 1775)
			***Sargocentron spiniferum *(Forsskål 1775)**
			*Sargocentron tiereoides* (Bleeker, 1853)
			*Sargocentron violaceum* (Bleeker, 1853)
	Gasterosteiformes		
		Aulostomidae	
			*Aulostomus chinensis* (Linnaeus, 1766)
		Fistulariidae	
			*Fistularia commersonii* (Rüppell, 1838)
		Pegasidae	
			***Eurypegasus draconis *(Linnaeus 1766)**
		Solenostomidae	
			***Solenostomus cyanopterus *Bleeker 1854**
			***Solenostomus halimeda *Orr, Fritzsche & Randall 2002**
			***Solenostomus paegnius *Jordan & Thompson 1914**
			***Solenostomus paradoxus *(Pallas 1770)**
		Syngnathidae	
			***Corythoichthys amplexus *Dawson & Randall 1975**
			***Corythoichthys haematopterus *(Bleeker 1851)**
			***Corythoichthys intestinalis *(Ramsay 1881)**
			***Corythoichthys ocellatus *Herald 1953**
			***Corythoichthys polynotatus *Dawson 1977**
			***Corythoichthys schultzi *Herald 1953**
			***Doryrhamphus dactyliophorus *(Bleeker, 1853)**
			*Hippocampus* sp.
			Sygnathidae sp.
			*Syngnathoides biaculeatus* (Bloch 1785)
			***Trachyrhamphus bicoarctatus *(Bleeker 1857)**
	Scorpaeniformes		
		Scorpaenidae	
			***Ablabys taenianotus *(Cuvier, 1829)**
			***Dendrochirus brachypterus *(Cuvier, 1829)**
			***Dendrochirus zebra *(Cuvier, 1829)**
			*Pterois antennata* (Bloch, 1787)
			*Pterois volitans* (Linnaeus, 1758)
			***Rhinopias aphanes *Eschmeyer, 1973**
			*Scorpaenodes albaiensis* (Evermann & Seale, 1907)
			*Scorpaenodes guamensis* (Quoy and Gaimard, 1824)
			*Scorpaenodes hirsutus* (Smith, 1957)
			*Scorpaenodes parvipinnis* (Garrett, 1864)
			*Scorpaenodes* sp. 1
			*Scorpaenodes* sp. 2
			***Scorpaenopsis diabolus *(Cuvier, 1829)**
			***Scorpaenopsis macrochir *Ogilby, 1910**
			***Scorpaenopsis oxycephala *(Bleeker, 1849)**
			***Scorpaenopsis possi *Randall & Eschmeyer, 2001**
			***Scorpaenopsis venosa *(Cuvier, 1829)**
			*Sebastapistes* sp.
			*Sunagocia* sp.
			***Taenianotus triacanthus *Lacépède, 1802**
		Synanceia	
			***Synanceia verrucosa *Bloch & Schneider 1801**
			
		Platycephalidae	
			***Cymbacephalus beauforti *(Knapp 1973)**
	Perciformes		
		Serranidae	
			*Anyperodon leucogrammicus* (Valenciennes 1828)
			*Cephalopholis argus* Schneider 1801
			*Cephalopholis boenak* (Bloch 1790)
			*Cephalopholis leopardus* (Lacépède 1801)
			*Cephalopholis miniata* (Forsskål 1775)
			*Cephalopholis urodeta* (Forster 1801)
			*Cromileptes altivelis* (Valenciennes 1828)
			*Diploprion bifasciatum* Cuvier 1828
			***Epinephelus fasciatus *(Forsskål 1775)**
			***Epinephelus fuscoguttatus *(Forsskål 1775)**
			***Epinephelus maculatus *(Bloch 1790)**
			*Epinephelus merra* Bloch 1793
			*Epinephelus polyphekadion* (Bleeker 1849)
			*Grammistes sexlineatus* (Thunberg 1792)
			*Plectropomus laevis* (Lacépède 1801)
			*Plectropomus leopardus* (Lacépède 1802)
			*Pseudanthias fasciatus* (Kamohara 1954)
			*Pseudanthias hypselosoma* Bleeker 1878
			*Pseudanthias luzonensis* (Katayama & Masuda 1983)
			*Pseudanthias pleurotaenia* (Bleeker 1857)
			*Pseudanthias squamipinnis* (Peters 1855)
			*Pseudanthias tuka* (Herre & Montalban 1927)
		Pseudogramminae	
			*Pseudogramma polyacantha* (Bleeker 1856)
			*Suttonia lineata* Gosline 1960
		Cirrhitidae	
			*Cirrhitichthys aprinus* (Cuvier, 1829)
			*Cirrhitichthys falco* Randall, 1963
			***Cirrhitichthys oxycephalus *(Bleeker, 1855)**
			*Oxycirrhites typus* Bleeker, 1857
			*Paracirrhites arcatus* (Cuvier, 1829)
			*Paracirrhites forsteri* (Schneider, 1801)
		Priacanthidae	
			***Priacanthus hamrur *(Forsskål 1775)**
		Psuedochromidae	
			***Pictichromis aurifrons *(Lubbock 1980)**
			***Pseudochromis fuscus *Müller & Troschel 1849**
			*Pseudochromis marshallensis* Schultz 1953
			*Pseudochromis* sp.
		Plesiopidae	
			***Calloplesiops altivelis *(Steindachner 1903)**
			*Plesiops caeruleolineatus* Rüppell, 1835
		Apogonidae	
			***Apogon aureus *(Lacépède, 1802)**
			*Apogon crassiceps* Garman, 1903
			***Apogon cyanosoma *Bleeker 1853**
			*Apogon exostigma* (Jordan and Starks, 1906)
			*Apogon fraenatus* Valenciennes, 1832
			***Apogon fucata *(Cantor, 1849)**
			***Apogon kallopterus *Bleeker, 1856**
			*Apogon nigrofasciatus* Lachner, 1953
			***Apogon perlitus *Fraser and Lachner, 1985**
			*Apogon rhodopterus* Bleeker, 1852
			*Apogon* sp. 1
			*Apogon* sp. 2
			*Apogon* sp. 3
			***Archamia zosterophora *(Bleeker, 1856)**
			***Cheilodipterus alleni *Gon, 1993**
			***Cheilodipterus isostigmus *(Schultz, 1940)**
			*Cheilodipterus macrodon* (Lacépède, 1802)
			*Cheilodipterus parazonatus* Gon, 1993
			*Cheilodipterus quinquelineatus* Cuvier, 1828
			*Cheilodipterus* sp*.*
			*Fowleria marmorata* (Alleyne and MacLeay, 1877)
			*Fowleria variegata* (Valenciennes, 1832)
			***Pseudamia hayashii *(Lachner & Fraser, 1985)**
			***Rhabdamia cypselurus *(Weber, 1909)**
			*Siphamia elongata* Lachner, 1953
			***Siphamia versicolor *(Smith & Radcliffe, 1911)**
			***Sphaeramia nematoptera *(Bleeker, 1856)**
			***Sphaeramia orbicularis *(Cuvier, 1828)**
		Carangidae	
			***Carangoides plagiotaenia *Bleeker, 1857**
			*Caranx melampygus* Cuvier, 1833
			*Caranx sexfasciatus* Quoy and Gaimard, 1825
		Lutjanidae	
			***Lutjanus argentimaculatus *(Forsskål, 1775)**
			*Lutjanus biguttatus* (Valenciennes, 1830)
			*Lutjanus gibbus* (Forsskål, 1775)
			*Lutjanus semicinctus* Quoy and Gaimard, 1824
			*Macolor macularis* Fowler, 1931
			***Symphorichthys spilurus *(Günther, 1874)**
		Caesionidae	
			*Caesio caerulaurea* Lacépède, 1801
			*Caesio cuning* (Bloch, 1791)
			*Caesio teres* Seale, 1906
			*Pterocaesio digramma* (Bleeker, 1864)
			*Pterocaesio pisang* (Bleeker,1853)
		Haemulidae	
			*Plectorhinchus chaetodontoides* Lacépède 1801
			*Plectorhinchus chrysotaenia* (Bleeker, 1855)
			*Plectorhinchus lineatus* (Linnaeus, 1758)
			*Plectorhinchus vittatus* (Linnaeus, 1758)
		Sciaenidae	
			*Sciaenops* sp.
		Lethrinidae	
			*Lethrinus erythracanthus* Valenciennes, 1830
			*Lethrinus harak* (Forsskål, 1775)
			*Lethrinus variegatus* (Valeciennes, 1830)
			*Monotaxis grandoculis* (Forsskål, 1775)
		Nemipteridae	
			*Pentapodus trivittatus* (Bloch, 1791)
			*Scolopsis bilineata* (Bloch 1793)
			***Scolopsis ciliatus *(Lacépède, 1802)**
			*Scolopsis lineata* Quoy & Gaimard 1824
			***Scolopsis margaritifera *(Cuvier 1830)**
			***Scolopsis monogramma *(Cuvier, 1830**)
		Mullidae	
			*Parupeneus barberinoides* (Bleeker, 1852)
			*Parupeneus crassilabris* (Valenciennes, 1831)
			*Parupeneus indicus* (Shaw, 1803)
			*Parupeneus multifasciatus* (Quoy and Gaimard, 1852)
			***Upeneus tragula *Richardson, 1846**
		Pempheridae	
			***Parapriacanthus ransonneti *Steindachner, 1870**
		Kyphosidae	
			*Kyphosus cinerascens* (Forsskål 1775)
		Chaetodonitdae	
			*Chaetodon auriga* Forsskål, 1775
			*Chaetodon baronessa* Cuvier, 1829
			*Chaetodon bennetti* Cuvier, 1831
			*Chaetodon citrinellus* Cuvier, 1831
			*Chaetodon ephippium* Cuvier, 1831
			*Chaetodon kleinii* Bloch, 1790
			*Chaetodon lunulatus* Quoy and Gaimard, 1825
			*Chaetodon melannotus* Bloch and Schneider, 1801
			*Chaetodon ornatissimus* Cuvier, 1831
			*Chaetodon pelewensis* Kner, 1868
			*Chaetodon plebeius* Cuvier, 1831
			*Chaetodon rafflesi* [Bennett], 1830
			*Chaetodon speculum* Cuvier, 1831
			*Chaetodon trifascialis* Quoy and Gaimard, 1825
			*Chaetodon ulietensis* Cuvier, 1831
			*Chaetodon unimaculatus* Bloch, 1787
			*Chaetodon vagabundus* Linnaeus, 1758
			*Chelmon rostratus* (Linnaeus, 1758)
			*Forcipiger flavissimus* Jordan and McGregor, 1898
			*Forcipiger longirostris* (Broussonet, 1782)
			*Hemitaurichthys polylepis* (Bleeker, 1857)
			*Heniochus acuminatus* (Linnaeus, 1758)
			*Heniochus chrysostomus* Cuvier, 1831
			*Heniochus singularis* (Smith and Radcliffe, 1911)
			*Heniochus varius* (Cuvier, 1829)
		Pomacanthidae	
			*Apolemichthys trimaculatus* (Cuvier 1831)
			*Centropyge bicolor* (Cuvier 1831)
			*Centropyge bispinosa* (Günther 1860)
			*Centropyge vrolikii* (Bleeker 1853)
			*Genicanthus melanospilos* (Bleeker 1857)
			*Pomacanthus imperator* (Bloch 1787)
			*Pomacanthus sexstriatus* (Cuvier 1831)
			*Pomacanthus xanthometopon* (Bleeker 1853)
			*Pygoplites diacanthus* (Boddaert 1772)
		Pomacentridae	
			*Abudefduf lorenzi* Hensley & Allen 1977
			*Abudefduf sexfasciatus* (Lacépède 1801)
			*Abudefduf vaigiensis* (Quoy & Gaimard 1825)
			*Amblyglyphidodon aureus* (Cuvier 1830)
			*Amblyglyphidodon curacao* (Bloch 1787)
			*Amblyglyphidodon leucogaster* (Bleeker 1847)
			*Amphiprion clarkii* (Bennett 1830)
			*Amphiprion melanopus* Bleeker 1852
			*Amphiprion percula* (Lacépède 1802)
			*Amphiprion perideraion* Bleeker 1855
			*Amphiprion polymnus* (Linnaeus 1758)
			*Chromis amboinensis* (Bleeker 1871)
			*Chromis atripectoralis* Welander & Schultz 1951
			*Chromis atripes* Fowler & Bean 1928
			*Chromis margaritifer* Fowler 1946
			*Chromis retrofasciata* Weber 1913
			*Chromis ternatensis* (Bleeker 1856)
			*Chromis viridis* (Cuvier 1830)
			*Chromis weberi* Fowler & Bean 1928
			*Chrysiptera rollandi* (Whitley 1961)
			*Chrysiptera talboti* (Allen 1975)
			*Dascyllus aruanus* (Linnaeus 1758)
			*Dascyllus melanurus* Bleeker 1854
			*Dascyllus reticulatus* (Richardson 1846)
			*Dascyllus trimaculatus* (Rüppell 1829)
			*Dischistodus chrysopoecilus* (Schlegel & Müller 1839)
			*Dischistodus prosopotaenia* (Bleeker 1852)
			*Neoglyphidodon melas* (Cuvier 1830)
			*Neoglyphidodon nigroris* (Cuvier 1830)
			*Neoglyphidodon oxyodon* (Bleeker 1858)
			*Neopomacentrus azysron* (Bleeker 1877)
			*Neopomacentrus taeniurus* (Bleeker 1856)
			*Plectroglyphidodon lacrymatus* (Quoy & Gaimard 1825)
			*Pomacentrus amboinensis* Bleeker 1868
			*Pomacentrus armillatus* Allen 1993
			*Pomacentrus bankanensis* Bleeker 1854
			*Pomacentrus cf. amboinensis* Bleeker, 1868
			*Pomacentrus cf. wardi* Whitley 1927
			*Pomacentrus colini* Allen 1991
			*Pomacentrus grammorhynchus* Fowler 1918
			*Pomacentrus moluccensis* Bleeker 1853
			*Pomacentrus nagasakiensis* Tanaka 1917
			*Pomacentrus nigromanus* Weber 1913
			*Pomacentrus pavo* (Bloch 1787)
			*Pomacentrus reidi* Fowler & Bean 1928
			*Premnas biaculeatus* (Bloch 1790)
			*Stegastes albifasciatus* (Schlegel & Müller 1839)
			*Stegastes fasciolatus* (Ogilby 1889)
			*Stegastes nigricans* (Lacépède 1802)
		Labridae	
			*Anampses neoguinaicus* Bleeker, 1878
			***Bodianus anthioides *(Bennet, 1832)**
			*Bodianus axillaris* (Bennet, 1832)
			***Bodianus bimaculatus *Allen, 1973**
			***Bodianus diana *(Lacépède, 1801)**
			*Bodianus mesothorax* (Bloch and Schneider, 1801)
			*Cheilinus chlorourus* (Bloch, 1791)
			*Cheilinus fasciatus* (Bloch, 1791)
			*Cheilinus oxycephalus* Bleeker 1853
			*Cheilinus trilobatus* Lacépède, 1801
			*Cheilinus undulatus* Rüppell, 1835
			*Choerodon anchorago* (Bloch, 1791)
			*Cirrhilabrus punctatus* Randall and Kuiter, 1989
			*Coris batuensis* (Bleeker, 1856–57)
			*Coris gaimard* (Quoy and Baimard, 1824)
			*Epibulus insidiator* (Pallas, 1770)
			*Gomphosus varius* Lacépède, 1801
			*Halichoeres argus* (Bloch and Schneider, 1801)
			*Halichoeres biocellatus* Schutlz, 1960
			*Halichoeres chloropterus* (Bloch, 1791)
			*Halichoeres hortulanus* (Lacépède, 1801)
			*Halichoeres leucurus* (Walbaum, 1792)
			*Halichoeres melanurus* (Bleeker, 1851)
			*Halichoeres prosopeion* (Bleeker, 1853)
			*Halichoeres richmondi* Fowler and Bean, 1928
			*Halichoeres trimaculatus* (Quoy and Gaimard, 1834)
			*Hemigymnus fasciatus* (Bloch, 1792)
			*Hemigymnus melapterus* (Bloch, 1791)
			*Hologymmnosus annulatus* (Lacépède, 1801)
			*Labrichthys unilineatus* (Guichenot, 1847)
			*Labroides dimidiatus* (Valenciennes, 1839)
			*Labropsis micronesica* Randall, 1981
			*Macropharyngodon meleagris* (Valenciennes, 1839)
			*Novaculichthys taeniourus* (Lacépède, 1801)
			*Oxycheilinus bimaculatus* (Valenciennes 1840)
			*Oxycheilinus digramma* (Lacépède, 1801)
			*Pseudocheilinus evanidus* Jordan and Evermann, 1903
			***Pseudocheilinus octotaenia *Jenkins, 1901**
			*Pseudocheilinus* sp.
			*Stethojulis bandanensis* (Bleeker, 1851)
			*Thalassoma hardwicke* (Bennett, 1830)
			*Thalassoma lunare* (Linnaeus, 1758)
			*Thalassoma lutescens* (Lay and Bennett, 1839)
			*Wetmorella nigropinnata* (Seale, 1901)
		Scaridae	
			*Calotomus carolinus* (Valenciennes 1840)
			*Calotomus spinidens* (Quoy & Gaimard 1824)
			***Cetoscarus bicolor *(Rüppell 1829)**
			*Chlorurus bleekeri* (de Beaufort 1940)
			*Chlorurus microrhinos* (Bleeker 1854)
			*Chlorurus sordidus* (Forsskål 1775)
			*Hipposcarus longiceps* (Valenciennes 1840)
			*Leptoscarus vaigiensis* (Quoy & Gaimard 1824)
			*Scarus chameleon* Choat & Randall 1986
			*Scarus flavipectoralis* Schultz 1958
			*Scarus frenatus* Lacépède 1802
			*Scarus ghobban* Forsskål 1775
			*Scarus niger* Forsskål 1775
			*Scarus quoyi* Valenciennes 1840
			*Scarus rivulatus* Valenciennes 1840
			*Scarus schlegeli* (Bleeker 1861)
			*Scarus spinus* (Kner 1868)
		Pinguipedidae	
			***Parapercis clathrata *Ogilby, 1910**
			*Parapercis hexophtalma* (Cuvier 1829)
			***Parapercis lineopunctata *Randall, 2003**
			*Parapercis millepunctata* (Günther, 1860)
			*Parapercis xanthozona* (Bleeker, 1849)
		Trichonotidae	
			***Trichonotus setiger *Bloch & Schneider 1801**
		Tripterygiidae	
			*Enneapterygius* sp.
			*Helcogramma* sp. 1
			*Helcogramma* sp. 2
			***Helcogramma striatum *Hansen, 1986**
		Blenniidae	
			*Aspidontus taeniatus* Quoy and Gaimard, 1834
			*Blenniella cf. gibbifrons* (Quoy and Baimard, 1824)
			*Crossosalarias macrospilus* Smith-Vaniz and Springer, 1971
			*Ctenogobiops* sp.
			***Ecsenius namiyei *(Jordan and Evermann, 1902)**
			*Ecsenius yaeyamaensis* (Ayoagi, 1954)
			***Meiacanthus grammistes *(Valenciennes, 1836)**
			***Meiacanthus vittatus *Smith-Vaniz, 1976**
			***Plagiotremus laudandus *(Whitley, 1961)**
			*Plagiotremus rhinorhynchos* (Bleeker, 1852)
		Gobiesocidae	
			***Diademichthys lineatus *(Sauvage, 1883)**
			***Discotrema crinophila *Briggs, 1976**
		Callionymidae	
			***Callionymus enneactis *Bleeker, 1879**
			***Dactylopus dactylopus *(Valenciennes, 1837)**
			***Synchiropus stellatus *Smith, 1963**
		Gobiidae	
			***Amblyeleotris arcupinna *Mohlmann and Munday, 1999**
			***Amblyeleotris guttata *(Fowler, 1938)**
			***Amblyeleotris randalli *Hoese and Steene, 1978**
			*Amblygobius decussatus* (Bleeker, 1855)
			*Amblygobius phaelena* (Valenciennes, 1837)
			***Amblygobius rainfordi *Whitley, 1940**
			***Bryaninops amplus *Larson, 1985**
			***Bryaninops loki *Larson, 1985**
			*Calumia* sp. 1
			*Calumia* sp. 2
			*Cryptocerus* sp.
			*Eviota* sp*.*
			*Exyrias belissimus* (Smith, 1959)
			***Fusigobius inframaculatus *(Randall, 1994)**
			***Fusigobius signipinnis *Hoese & Obika 1988**
			***Fusigobius *sp.**
			Gobidae sp. 1
			Gobidae sp. 2
			Gobidae sp. 3
			***Gobiodon okinawae *Sawada, Arai & Abe, 1972**
			*Istigobius goldmanni* (Bleeker, 1852)
			*Istigobius ornatus* (Rüppell, 1830)
			***Istigobius rigilius *(Herre, 1953)**
			***Oplopomus oplopomus *(Valenciennes, 1837)**
			Oxudercinae sp.
			***Paragobiodon xanthosomus *(Bleeker, 1852)**
			*Periophthalmus argentilineatus* Valenciennes, 1837
			***Pleurosicya bilobata *(Koumans, 1941)**
			***Pleurosicya micheli *Fourmanoir, 1971**
			***Pleurosicya mossambica *Smith, 1959**
			*Priolepis* sp.
			*Signigobius biocellatus* Hoese & Allen 1977
			*Trimma* sp. 1
			*Trimma* sp. 2
			*Trimma* sp. 3
			***Trimma caesiura *Jordan & Seale 1906**
			*Trimma macrophthalma* (Tomiyama, 1936)
			*Trimma okinawae* (Aoyagi, 1949)
			*Trimma striatum* (Herre 1945)
			***Valenciennea helsdingenii *(Bleeker 1858)**
			*Valenciennea puellaris* (Tomiyama 1956)
			*Valenciennea strigata* (Broussonet, 1782)
		Xenisthmidae	
			*Xenisthmus cf. polyzonatus* (Klunzinger 1871)
		Ptereleotridae	
			***Nemateleotris decora *Randall & Allen 1973**
			*Nemateleotris magnifica* Fowler 1938
			*Ptereleotris evides* (Jordan & Hubbs 1925)
		Ephippidae	
			*Platax orbicularis* (Forsskål, 1775)
			***Platax pinnatus *(Linnaeus, 1758)**
			*Platax teira* (Forsskål, 1775)
		Zanclidae	
			*Zanclus cornutus* (Linnaeus 1758)
		Acanthuridae	
			*Acanthurus auranticavus* Randall, 1956
			*Acanthurus fowleri* de Beaufort, 1951
			*Acanthurus grammoptilus* Richardson, 1843
			*Acanthurus lineatus* Linnaeus, 1758
			*Acanthurus nigrofuscus* (Forsskål, 1775)
			*Acanthurus nigroris* Valenciennes, 1835
			*Acanthurus olivaceus* Bloch and Schneider, 1801
			*Acanthurus pyroferus* Kittlitz, 1834
			*Acanthurus triostegus* (Linnaeus, 1758)
			*Ctenochaetus binotatus* Randall, 1955
			*Ctenochaetus striatus* (Quoy and Baimard, 1825)
			*Naso brevirostris* (Cuvier, 1829)
			*Naso lituratus* (Forster, 1801)
			*Naso vlamingii* (Valenciennes, 1835)
		Siganidae	
			*Siganus argenteus* (Quoy & Gaimard 1825)
			***Siganus javus *(Linnaeus 1766)**
			*Siganus puellus* (Schlegel 1852)
			*Siganus spinus* (Linnaeus 1758)
			*Siganus vulpinus* (Schlegel & Müller 1845)
		Sphyraenidae	
			***Sphyraena flavicauda *Rüppell 1838**
			*Sphyraena qenie* Klunzinger 1870
		Scombridae	
			*Euthynnus affinis* (Cantor 1849)
			*Katsuwonus pelamis* (Linnaeus 1758)
			*Rastrelliger kanagurta* (Cuvier 1816)
			*Scomberoides lysan* (Forsskål 1775)
			*Scomberoides tol* (Cuvier 1832)
	Pleuronectiformes		
		Bothidae	
			***Bothus mancus *(Broussonet, 1782)**
		Soleidae	
			***Pardachirus pavoninus *(Lacépède 1802).**
			*Pardachirus* sp.
	Tetraodontiformes		
		Balistidae	
			*Abalistes stellatus* ([Lacépède, 1798])
			*Balistapus undulatus* (Park, 1797)
			*Balistoides conspicillum* (Bloch and Schneider, 1801)
			*Balistoides viridescens* (Bloch and Schneider, 1801)
			*Melichthys vidua* (Richardson, 1845)
			*Pseudobalistes flavimarginatus* (Rüppell, 1829)
			*Rhinecanthus aculeatus* (Linnaeus, 1758)
			*Rhinecanthus verrucosus* (Linnaeus, 1758)
			*Sufflamen bursa* (Bloch and Schneider, 1801)
			*Sufflamen chrysopterus* (Bloch and Schneider, 1801)
		Monacanthidae	
			*Aluterus scriptus* (Osbeck 1765)
			*Cantherhines dumerilii* (Hollard 1854)
			*Cantherhines pardalis* (Rüppell 1837)
			*Monacanthus chinensis* (Osbeck, 1765)
			*Oxymonacanthus longirostris* (Bloch & Schneider, 1801)
			*Pervagor cf. melanocephalus* (Bleeker, 1853)
			*Pervagor janthinosoma* (Bleeker, 1854)
			*Rudarius minutus* Tyler, 1970
		Ostraciidae	
			*Lactoria cornuta* (Linnaeus, 1758)
			*Ostracion cf. cubicus* Linnaeus, 1758
			*Ostracion meleagris* Shaw, 1796
			*Ostracion solorensis* Bleeker, 1853
		Tetraodontidae	
			*Arothron caeruleopunctatus* Matsuura 1994
			*Arothron hispidus* (Linnaeus 1758)
			*Arothron manilensis* (Marion de Procé 1822)
			*Arothron mappa* (Lesson 1831)
			*Arothron nigropunctatus* (Bloch & Schneider 1801)
			*Arothron stellatus* (Anonymous 1798)
			*Canthigaster compressa* (Marion de Procé 1822)
			*Canthigaster janthinoptera* (Bleeker, 1855)
			*Canthigaster papua* (Bleeker 1848)
			*Canthigaster valentini* (Bleeker 1853)
			Tetraodontidae sp.
		Diodontidae	
			*Diodon hystrix* Linnaeus, 1758

Phylogenetic classification after [44]. Species in **BOLD** were not observed during the January 2011 survey, but reported in the literature or in museum collections Species were identified to the finest taxonomic resolution possible.

### Species richness

We used two different methods to generate species richness estimates, using a combined data set that includes both our field-based sampling records and reports from the literature. We first calculated Allen’s [11] Coral Fish Diversity Index (CFDI), which is the sum of species numbers in the Acanthuridae, Chaetodontidae, Pomacanthidae, Pomacentridae, Labridae and Scaridae families. The CDFI value for Bootless Bay is 158, which when used to estimate total number of fish species in the region yields values between 515 and 558.

We also used an incident-based methodology that specifically calculates species richness based on presence-absence and not density of species [12,13]. The results from the incident-based estimators resolved consistently larger values, with the Incident-based Coverage Estimator (ICE) estimating 949 species and the Chao 2 estimating 939 species (95% confidence: 767–74 to 1187 species).

## Discussion

Papua New Guinea lies within the Coral Triangle, the global epicenter of marine biodiversity. While the mechanisms underlying the Coral Triangle epicenter of diversity phenomenon remain to be fully explored [14-17], the pattern itself has been reported for well over a century [18]. Different analytical methods used to estimate species number in Bootless Bay have resulted in varying species estimates. Our species CFDI estimates (n = 515-558) are substantially lower than those estimated for other areas in PNG (n > 800, [11]). More data-rich ecological estimates resulted in approximately 940 species in Bootless Bay. We suggest that the latter estimates are more in line with the true species number, as the CFDI extrapolates total diversity from several surrogate families, while the ICE and Chao 2 estimators incorporate a richer data set by generating estimates based on all species encountered. The CFDI may be disproportionably impacted by fisheries pressure as it relies on families of fish, such as Scarids (parrotfish) and Labridae (wrasses), which contain several economically valuable species. Therefore species richness estimates based on the CFDI may be highly sensitive to fisheries pressure. However we retain the CFDI measurements in order to be able to make our data set comparable with other published literature.

In absolute numbers the ichthyofauna of Bootless Bay is diverse, with more species of reef fish present than in Belize (n = 369), Kiribati (n = 426), the Bahamas (n = 457), or the Cook Islands (n = 477) [19], but see [20]. Despite the large absolute number of species, the relative species composition of Bootless Bay, when compared to other sites in Papua New Guinea, is rather depauperate. Allen et al… [11] reported species numbers of 1313 reef fish for Milne Bay and 850 for Madang, while Munday et al… list 881 for Kimbe Bay, PNG, using the more conservative CFDI methodology (pers. com with Phil Munday, James Cook University for reference our Bootless Bay value using the same methodology estimated 515–558 species). Other species lists for sites in Papua Province in the Republic of Indonesia include 1511 species for the Bird’s Head peninsula as a whole [21] and 1357 from Raja Ampat alone [22]. We urge caution in making direct comparisons among these regions, as sampling effort is unequal, disproportionately influencing low diversity areas such as Bootless Bay. As additional surveys are carried out within Bootless Bay we anticipate an increase in the diversity of species recorded.

Bootless Bay differs in habitat complexity from other regions of Papua New Guinea, which could contribute to the differences seen in species numbers. While Milne Bay and Kimbe Bay contain a wide diversity of habitats [11,23], Bootless Bay has relatively low complexity with fewer habitat types (Allen pers. com.), suggesting that habitat availability could be a contributing factor to differences in species diversity.

Bootless Bay’s reefs lie less than 10 km from Port Moresby, a city with an urban population of over 300,000, the largest in Melanesia. A large portion of this population comes from internal migration from rural areas into Port Moresby. Because of this influx, the marine resources of Bootless Bay are increasingly used to provide protein for this rapidly growing urban population [24]. Port Moresby has been the country’s major population center since colonial times, and the impacts of its population on local reefs, while certainly larger than historical levels, are by no means new occurrences.

Along with primary resource use, the reefs of Bootless Bay are also influenced by siltation from freshwater sources. The quality of these inflows has been severely impacted due to upstream conversion of primary and secondary forests into agricultural land coupled with the unplanned urban growth around squatter villages [10,25]. It is likely that the proximity of these reefs to the country’s population center, and the resource exploitation and habitat degradation that proximity entails, also contribute to the low species diversity in Bootless Bay.

A recent study [26] examining the percentage of living coral cover at four stations located on fringing and patch reefs reported a steady decline in coral cover that correlated with distance from the Bootless Bay coastline. Although a correlation between percentage coral cover and sediment levels was not significant due to the small sample size, a detailed study is required to better understand the impact of siltation on living coral decline in the bay and how this can contribute to habitat loss and a reduction in fish diversity. Furthermore, this will allow for appropriate management decisions to be made regarding construction projects and land use practices in nearby areas.

### Conservation in Bootless Bay

The reefs of Papua New Guinea face a suite of threats from local impacts (over-fishing, development, siltation), transnational (shifts in fisheries pressure, live reef fish trade) and global sources (increased sea surface temperatures, oceanic acidification) [8,27,28]. Despite these threats, the reefs still house a high diversity of fishes and are a critical national resource for hundreds of thousands of people. Proper management of these reefs depends on first identifying the state of the reefs and then taking actions to mitigate threats to them.

The results presented here regarding fish biodiversity represent an important step in identifying the state of the reefs of Bootless Bay. While we acknowledge that our species list is incomplete, listing the species living here in 2011 establishes a baseline of fish diversity that is necessary for future conservation action. Tracking species’ presence and absence is an important way to monitor ecosystems, and future surveys that fail to detect species present in our list will suggest a further degradation of these reefs. Additional studies that record species abundances as well as species richness would complement our study and enrich the conservation utility of ichthyologic surveys. Recording abundance of several key fisheries species which are indicative of healthy reefs, including large groupers (*Epinephelus polyphekadion, Plectropomus leopardus*), jacks (*Caranx melampygus*) and sharks (*Carcharhinus melanopterus*), may provide more fine scaled environmental monitoring than simply presence or absence data [29]. Similarly tracking changes in parrotfish abundance will allow for monitoring of ecological important guilds, the removal of which can have drastic changes to reef functioning [30]. Additionally, by placing emphasis on large, easily identified species, one is able to leverage citizen scientists to help monitor changes in reef quality. Such recreational diver surveys have been helpful in tracing large scale biodiversity patterns in the Caribbean [31] and the Pacific [32].

Effective conservation of reef resources often requires a multifaceted approach that includes a mixture of no-take zones, sustainable economic development and local community participation [8,33,34]. Bootless Bay has all of these necessary components, including a small no-take reserve around Motupore Island, an ecologically-minded dive resort that requires healthy reefs for its livelihood and, through the auspices of the University of Papua New Guinea, a cadre of educated and well-trained local conservation practitioners. The expansion of the marine protected area (MPA) and subsequent increased educational, employment and monitoring opportunities would provide additional protection for these reefs, which in turn could potentially benefit the local tourism economy. In theory a small “environmental health” tax levied on divers could potentially offset the cost of running the reserve [35,36]. However, it is important to note that the effectiveness of an MPA is dependent on the cultural context within which it is enacted, and we caution against coarse grain conservation measures that do not involve local stakeholder participation [27,37,38].

### Bioinformatic resources

In a recent review Drew [39] highlighted the role that bioinformatic resources can play in conserving biodiversity. A major point was the ability of on-line resources to facilitate research countries that are rich in biodiversity but poor in conservation resources. In this spirit we have chosen to publish our work here, in an open-access journal, so that the people who are most in need of these data are not limited in their access to them. We have also published the species list in Dryad, an international, freely accessible, data depository site ( http://datadryad.org) to facilitate the wide distribution of our data. It may be accessed with the http://html://dx.doi.org/10.5061/dryad.k2v04.

We also envision this checklist serving as a living document that has an updated list augmented annually as new species are described or identified or as existing taxonomies are modified. In addition, we will work with other researchers in the region to maintain a comprehensive record of species as they are observed. This checklist is an excellent avenue to engage citizen scientists in monitoring. By encouraging submission from recreational divers, snorkels and anglers we are able to incorporate a more thorough temporal and spatial sampling regime that complements existing synoptic surveys. Similar programs have been instrumental in recording shifts in species abundance brought about by climate change [30] and in helping to describe subtle shifts in community structure [40]. This type of dynamic publishing would not have been possible as little as five years ago, and we encourage other researchers to follow this model and make their data as broadly accessible as possible.

## Conclusions

In summary we present a list of 485 species of marine fishes found in the Bootless Bay region of Papua New Guinea. We use these data to extrapolate a total species richness of approximately 940 species. The species richness of Bootless Bay is lower than other reports for reefs around the island of New Guinea (including those in Papua New Guinea and the Indonesian province of West Papua). This lower species number is probably a combination of natural (lower habitat complexity) and anthropogenic (fisheries pressure, upland habitat modification) stressors. Further sampling in the region will undoubtedly result in additional species being recorded for the area. However, the major contribution of the present work is to clearly delineate, both spatially and temporally, the marine fish biodiversity of reefs of Bootless Bay. Moreover we present a detailed methodology so that future researchers can produce directly comparable datasets.

## Methods

### Specimen collection

Specimens were collected from January 15-27^th^, 2011, as part of a joint Field Museum of Natural History and University of Papua New Guinea expedition. We used a variety of methods to obtain specimens including rotenone stations, spear fishing, fish market purchases, hand line fishing and in some cases capture of samples by hand. All necessary permits and permissions were obtained from the University of Papua New Guinea (which manages the Motupore Island Research Station) and the PNG Department of Environment and Conservation (the relevant regulatory body concerned with protection of wildlife), and all collections were made with the permission of and in accordance with the laws of Papua New Guinea and the United States as well as all applicable international treaties.

For rotenone stations [41] we identified a small (2 m) tabular coral (usually *Acropora* sp.) that was isolated by at least 2 m of sand. The depth of each station varied between 3–32 m, all within safe SCUBA depth. Approximately 1 kg of rotenone mixed with 1 l of saltwater and a small portion of dish soap to act as an emulsifier was combined and distributed *in situ* over the surface of the tabular coral by one diver. Two to four additional individuals positioned themselves 1–2 m above the initial rotenone ‘cloud’ to capture larger fishes escaping. After an initial period of ~10 min all divers descended to the bottom and searched in expanding circles for fish that had succumbed to the effects of rotenone. Collections typically took 2 person/h.

For spear fishing stations we targeted fishes along isolated patch reefs or a section of barrier reef. Sampling individual fishes in this way maximizes diversity and minimizes the ecological impact of collecting. Because larger predatory fishes were extremely rare (e.g., only two individual sharks were spotted despite 120 hours of diving), we chose to record but not collect large members of Serranidae (e.g., *Plectropomus laevis, P. leopardus*), Carangidae (e.g., *Caranx melampygus*) and Carcharhinidae (*Carcharhinus melanopterus*). Collecting effort as measured by raw number of individuals decreased as spear sampling effort increased simply because we collected common species early. However, the number of new species collected continued to increase even up until the final spear fishing station (2 new species collected at Station 33 - Table 1).

We also collected specimens from one of four large fish markets in Port Moresby. The market was stocked by ~40 individual retailers, and from interviewing them we found that most fishing was done with hand lines or nets on small boats driven by <60 hp engines. Because this was an active market all species were, by definition, commercially exploited. We saw several species for sale at the market that were not found during our collections (e.g. *Caranx sexfasciatus*, *Megalops cyprinoids*, *Moolgarda seheli*, *Rastrelliger kanagurta* and *Euthynnus affinis*). The presence of *R. kanagurta* and *E. affinis* suggests that the fishers were expanding their effort to offshore, non-reef areas, although the power of the boats’ engines and lack of refrigeration onboard probably precludes the fishers from traveling too great a distance from the region. Port Moresby lies on a shallow continental margin, but because the shelf break occurs relatively close (Figure 1) at about 135–140 m, fishers from the region have easy access to open water species [42].

Individual fish were photographed and identified to species level within two hours of collecting. Most individuals had small pieces of muscle or gill tissue subsampled and preserved in 95% EtOH for future DNA analysis. All specimens were then fixed in formalin or skeletonized and ultimately deposited in the collections of the Field Museum of Natural History. Field identifications were later validated or revised in the laboratory using keys and the Field Museum’s reference collections. Current taxonomic rank assignment, valid names and species distributions were evaluated using FishBase [19], the Encyclopedia of Life ( http://www.eol.org) and Randall [43,44]. In our total species list we also included data from Baine and Harasti [45] and on-line museum collections accessed through http://www.fishnet2.net with the “Search Polygon” feature centered around Bootless Bay.

### Species accumulation analysis

We used EstimateS 8.2 [13] to generate a species accumulation curve (or sample-based rarefaction curve *sensu*[46]) for our field based sampling. We first randomized our sampling sites with 50 randomizations and then generated the Mau Tau richness function and the associated 95% confidence intervals [13,46].

### Species richness analysis

To calculate estimates of total species number, we used a combined data set that includes both our collections records and reports from the literature and generated Allen’s [11] Coral Fish Diversity Index (CFDI). This index is the sum of species numbers in Acanthuridae, Chaetodontidae, Pomacanthidae, Pomacentridae, Labridae and Scaridae. The CDFI can then be used to generate an estimate of total species number for the larger region (>50,00 km^2^) and the specific area (<2000 km^2^) using the formulae

(1) 4.234 × CFDI - 114.446 = total fauna in a surrounding area greater than 50,000 km^2^

and

(2) 3.39 × CFDI - 20.595 = total fauna in a surrounding area less than 2000 km^2^[11].

Finally, we calculated two estimates of species richness using the software EstimateS 8.2 [13]: the Incidence -based Coverage Estimator (ICE) [12] and the non-parametric Chao 2 [47,48].

Both metrics share the advantage of basing estimates on presence/absence data while taking into account species not present in any samples [13]. They differ in the relative weights they place on rare species, with ICE being based on species found in 10 or fewer locations, with Chao 2 being driven more by the number of singletons or doubletons in the data set [49]. Because we tried to minimize the ecological impacts of our collecting, in general we did not collect more than a few individuals of any individual species, thus metrics that require abundance information to estimate species richness would be skewed by our collecting methodology.

## Competing interests

The authors declare that they have no competing interests.

## Authors’ contributions

Sampling strategy and trip logistics were designed by JD MW and AM. Samples were collected by JD MW JM and CB, with permitting help from AM. Species were identified by MW JD JM CB AR and DH, with species richness estimates performed by JD AR and DH. The manuscript was written by JD JM MW and AM. All authors read and approved the final manuscript.
